# “Everybody Puts Their Whole Life on Facebook”: Identity Management and the Online Social Networks of LGBTQ Youth

**DOI:** 10.3390/ijerph15061078

**Published:** 2018-05-26

**Authors:** Elizabeth McConnell, Bálint Néray, Bernie Hogan, Aaron Korpak, Antonia Clifford, Michelle Birkett

**Affiliations:** 1 Department of Psychology, DePaul University, 2219 N. Kenmore Ave., Chicago, IL 60614, USA; korpak@northwestern.edu; 2 Department of Medical Social Sciences, Feinberg School of Medicine, Northwestern University, 625 N. Michigan Ave., Suite 1400, Chicago, IL 60614; USA; balint.neray@northwestern.edu (B.N.); antonia.clifford@northwestern.edu (A.C.); birkett@northwestern.edu (M.B.); 3 Department of Sociology, University of Oxford, Manor Road Building, Manor Road, Oxford OX1 3UQ, UK; bernie.hogan@oii.ox.ac.uk

**Keywords:** LGBTQ youth, Facebook, outness, context collapse, social network analysis, network visualization

## Abstract

Lesbian, gay, bisexual, transgender, and queer (LGBTQ) youth and young adults almost inevitably “come out”, or self-disclose their identity to others. Some LGBTQ youth are more uniformly “out”, while others may disclose to some groups but not others. This selective disclosure is complicated on real name social media sites, which tend to encourage a unified presentation of self across social contexts. We explore these complications with a cohort of LBGTQ youth on Facebook (*N* = 199, M_age_ = 24.13). Herein we ask: How do LBGTQ youth manage the disclosure of their sexual orientation and/or gender identity to different people in their lives? Further, are there identifiable differences in the online social network structure for LGBTQ youth who manage outness in different ways? Finally, how do LGBTQ young people describe their experiences on Facebook? We answer these questions using a mixed methods approach, combining statistical cluster analysis, network visualization, and qualitative data. Our findings illustrate patterns in network structure by outness cluster type, highlighting both the work involved in managing one’s online identity as well as the costs to (semi-) closeted individuals including a considerably lower overall network connectivity. In particular, outness to family characterized LGBTQ young people’s experiences on Facebook.

## 1. Introduction

Social media is rapidly changing the landscape within which young people negotiate their lives, introducing new complications in the balance between public and private selves. Information which may previously have been shared with only a few close network members is now easily communicated to the farthest reaches of one’s Facebook network, which may be just as likely to include close friends as it is to include old classmates, casual acquaintances, and extended family members with strong political differences. This phenomenon of heightened interconnection and reduced social boundaries across diverse network subgroups, known as context collapse, has far-reaching implications for how young people negotiate their identities in online spaces [1,2,3,4,5]. It is of particular importance to LGBTQ young people, who are faced with the task of negotiating how they manage their sexual and gender identities on social media. However, the online contexts of LGBTQ young people have been understudied due to a focus on the experiences of members of dominant groups [6]. Further, research on LGBTQ experiences online has often relied on traditional methods like survey data, which provide an indirect and very general picture of participants’ online social worlds. By contrast, network approaches support the efficient collection and analysis of rich and detailed data about participants’ social media relationships. In the current study, we utilized a mixed methods approach integrating survey, network, and qualitative data to explore how LGBTQ young people experience their identities on Facebook. 

### 1.1. Online Identity Management

In 2010, Mark Zuckerberg, the co-creator and CEO of Facebook suggested that “having two identities for yourself is an example of a lack of integrity” [7]. While Zuckerberg was referring to those who hide behind pseudonyms in order to troll or defame, he inadvertently trivialized those who have no choice but to manage multiple identities. Beyond Zuckerberg’s quote is an extensive literature on how social media intentionally and unintentionally reveal different facets of a person’s identity to a single audience, which often includes diverse relational subgroups. People often play a variety of social roles (for example, a family member, a co-worker, a friend) across these relational subgroups, which may also have very different values and norms. Social media promotes interaction outside of the settings where these roles are typically played, which can create a number of identity management and information access concerns for users. This is particularly true for real name social media like Facebook as compared to more anonymous online contexts. 

In the computer-mediated communication literature, this phenomenon has been referred to as “context collapse”. Initially introduced by Boyd and her colleagues (cf., Marwick and Boyd, [8]), this concept has become central to understanding online identity expression. Vitak and Kim [9] illustrated how collapsed contexts often create significant impression management challenges that require individuals to think carefully about what they reveal online. Hogan [4] drew upon early conceptions of context collapse to assert that individuals strategically withhold information in order to create an online presentation that satisfies the “lowest common denominator” of one’s perceived online audience, which posits that individuals share information about themselves on Facebook only to the extent that they are comfortable with people who may find this information problematic seeing it. Zhao and colleagues [10] extended Hogan to show how individuals often update this lowest common denominator by reviewing past online content such as “wall posts” and photos. More recently, Davis and Jurgenson [11] have recast the concept in terms of *context collisions* (where different social groups are aggregated and identities spill over between them) and *context collusions* (where individuals deliberately bring disparate members of their social network together). The process of selective disclosure affects virtually everyone, as at its core it is about the construction of a private self that is revealed to some people and a public self that is assumed to be available to anyone, regardless of context. Yet for some individuals, this process of selective disclosure is especially salient. 

### 1.2. Identity Disclosure for LGBTQ Young People

Outside of the realm of social media, many LGBTQ young people already manage disclosure of their identities in complicated ways that have important implications for well-being. On the one hand, being “out” about one’s identity may help LGBTQ young people experience a greater sense of authenticity in their relationships, avoid the stresses associated with identity concealment, and access LGBTQ related social support from network members, thus, leading to improved well-being. On the other, identity disclosure in unsupportive contexts can lead to the loss of support and experiences of rejection or victimization, thus leading to diminished well-being. 

Research on identity disclosure among LGBTQ young people illustrates that its associations with well-being are complicated and context-dependent. Research has generally found identity concealment to be associated with greater depression and lower psychological well-being, while authenticity has been associated with lower depression and greater psychological well-being. However, research suggests these relationships depend on how supportive or rejecting the context is [12,13]. A national study of over 7800 LGBTQ high school students found that outness was related to greater victimization (which often adversely impacts well-being), but also to greater self-esteem and lower depression [14]. The study also found that contextual factors influenced these relationships, such that students in rural schools experienced more negative effects of being out than students in urban schools [14]. The role of context in shaping the relationships between outness and well-being has been underscored by other research, which found that disclosure was associated with well-being in relational contexts that promote interpersonal acceptance and authentic self-expression, but not in relational contexts where people are expected to act or perform a certain way [15]. Also, outness was associated with health benefits for men of higher socioeconomic status but health problems for men of lower socioeconomic status [16]. Thus, being “out” about one’s sexual or gender minority identity appears to be associated with well-being in some contexts, but not others.

LGBTQ young people manage identity disclosure within particular social roles and relational contexts (for example, school, work, family, neighborhood), the characteristics of which shape the likelihood and consequences of disclosure. For example, supportiveness was particularly important in shaping how sexual minority people managed identity disclosure within their families (relative to within their workplaces), such that individuals with both high and low outness were less likely to disclose to their families if they expected a negative reaction [17]. Similarly, sexual minority people of color were less likely to disclose their identities to their families if they experienced a “conflict of allegiance” between their racial/ethnic community and their LGBTQ community [18]. Research also found that the presence or absence of family support characterized the landscape of LGBTQ young people’s experiences of social support and psychological well-being [19,20], which suggests that family relationships, in particular, may be a salient context within which LGBTQ young people negotiate decisions about identity disclosure in order to maximize their psychological well-being. Overall, perceived acceptance, context, and individual differences in outness come together to shape disclosure decisions [17], meaning that disclosure can be particularly complex for LGBTQ young people whose relational contexts vary with respect to their degree of supportiveness and acceptance.

### 1.3. LGBTQ Identities Online

Given these complex relational landscapes, the phenomenon of online context collapse presents particular challenges for LGBTQ young people. To negotiate these disclosure-related challenges, LGBTQ young people engage in a variety of identity management strategies, including monitoring their online self-expression, using privacy and security controls, strategically managing their friendship networks, creating multiple accounts, curating and editing personal photographs, and restricting LGBTQ-related to other, more anonymous online contexts [2,21,22,23]. These strategies require a good deal of labor—both traditional labor with respect to time and effort and emotional labor with respect to self-presentation [9,10]. Further, privacy and security settings are often difficult to navigate and imperfect with respect to information control. One high profile example of these difficulties happened in 2012 in Texas, when two students were added to a public Facebook group for a lesbian and gay university choir, which inadvertently outed them to their entire Facebook networks without their consent. This inadvertent disclosure, in turn, led to conflict with unsupportive family members and negative mental health outcomes [24].

Given the complexity of these identity management negotiations in online contexts, a number of researchers have employed qualitative methods to better understand these processes among LGBTQ young people. Taylor and colleagues [25] examined how LGBTQ Christian youth negotiated their online and offline identities to manage potential conflicts between their religious and sexual identities; some participants used Facebook as a space to visibly embody both identities, while others strategically negotiated visibility in different spaces in order to manage potential conflicts between their sexual and religious identities and communities. A study of young queer women in San Francisco found that participants engaged in substantial emotional labor to negotiate the online visibility of their sexual identities to family, including engaging in perpetual surveillance of their Facebook presences and ruminating about profile content [26]. A study of young gay men in the Rocky Mountain West found that participants negotiated their sexual identities on Facebook in three distinct ways: by celebrating and affirming their identities; by indirectly coming out to some network members but not others; and by actively concealing their identities [27]. Finally, a study of diasporic gay men in Belgium (including both second-generation immigrants and sexual refugees) found that participants more openly expressed their sexual identities on gay-friendly social media sites outside of Facebook, as they offered greater discretion and safer opportunities for self-exploration [28].

Across all of these studies, whether or not families were supportive of LGBTQ young people’s sexual identities emerged as an important variable that shaped how they negotiated their identities on Facebook. Thus, although the challenges associated with identity management on Facebook are unique given the particular conditions of context collapse, these findings are, in many, ways consistent with research demonstrating the importance of supportive relational contexts (particularly families) in shaping how LGBTQ young people manage identity disclosure in their overall lives. 

### 1.4. Network Approaches

Although these qualitative studies have contributed a great deal to knowledge about identity management concerns for LGBTQ young people online, there is a lack of research on the specific relational contexts of LGBTQ young people. Facebook network data provides a great deal of valuable information about the online relational contexts of individuals, including the extent to which online networks reflect different forms of social capital [29,30,31]. Brooks and colleagues [32] noted that the relationships between social capital and network structure were slightly different for Facebook networks than for the other types of networks typically examined in social network analysis. For example, network transitivity (that is, the number of closed paths in a network) is believed to be associated with higher bonding social capital, given that it is typically understood to represent dense reciprocal relationships. However, lower transitivity was actually associated with higher bonding social capital in Facebook networks. The authors describe how transitivity may operate differently in Facebook networks because these networks often represent multiple social groups that may very well have reasons to overlap (for example, high school friends from one’s hometown and current co-workers). They argue that global cohesion (achieved through ties that bridge between network subgroups) is a better measure of bonding social capital in Facebook networks, and note that people with more cohesive Facebook networks likely experience online context collapse as less disruptive or distressing than those with less cohesive networks, who may experience a greater demand to negotiate information sharing to distinct network subgroups. 

Following from this, we would expect that LGBTQ young people who are not out to their entire network would not only have a harder time managing their online networks, but would also feel less connected to and reap lower social capital from their online networks. The cruel irony is that those who wish to segment their network are likely more vulnerable, as this segmentation is often used to protect against rejection and other adverse experience in non-affirming relational contexts. Thus, despite a potentially greater need for social support from their network, the imperative to keep social contexts separate might lead to lower social capital, greater impression management challenges, and even potentially greater feelings of isolation. Given the wealth of information Facebook network data can provide, research that incorporates and examines the network structure as an important aspect of the online relational contexts negotiated by LGBTQ young people is needed.

### 1.5. Current Study

In the current study, we used a mixed methods approach to examine identity management and the Facebook relational contexts of LGBTQ young people. We posed three research questions about the relationships between outness, Facebook network structure, and online identity management:

1. What patterns of outness to different relational contexts are demonstrated by LGBTQ youth? If we cluster LGBTQ young people on the basis of whether they are out to different relational subgroups, do we see distinct and interpretable clusters representing a typology?
2. What is the relationship between outness and Facebook network structure? When people are out to some relational subgroups but not others, is this associated with differences in network composition and/or connectivity between these groups?
3. How do LGBTQ young people describe their experiences on Facebook? Are these experiences different for those who manage outness in different ways?

By leveraging the strengths of the network, survey, and qualitative data, we aim to contribute a nuanced picture of how the young people in our study negotiated outness in their overall lives and, in turn, how this shaped their relationships and experiences on Facebook. Given the rapid increase in Facebook use among young adults in the past decade [33], we believe this provides a much-needed examination of the online social contexts of LGBTQ young people.

## 2. Methods

### 2.1. Participants and Procedures

Participants were a community sample of 204 youth and young adults aged 19 to 28 who currently or formerly lived in the Chicago area and self-identified as LGBT, queer, questioning, or same-gender attracted. Participants for this study were recruited from an ongoing longitudinal study of LGBTQ youth (see Reference [34]). Data collection was conducted in 2013 and 2014. In terms of demographics, 92 were assigned male at birth and 112 were assigned female at birth; 77 identified as male, 108 as female, 15 as transwomen, and 3 as transmen; 69 identified as gay, 55 as lesbian, 49 as bisexual, 10 as heterosexual, and 8 as questioning/unsure; 116 identified as African-American, 28 as White, 23 as Hispanic/Latino, 23 as multi-racial, and 14 as other. The mean age was 24.13 years (*SD* = 1.64). Further description of the study is reported elsewhere [35].

Participants first completed a number of survey measures, including a five-item measure of outness. Outness was assessed using an adaptation of the Outness Inventory [15,36], which asked participants to rate their outness on a scale of 1 (*not at all out*) to 5 (*completely out*) to each of the following subgroups: family, friends, classmates, co-workers, and others in general. Participants were asked separately about overall (α = 0.90) and online (α = 0.95) outness. 

Following the survey measures, participants were asked a series of open-ended questions about how they used Facebook and their positive and negative experiences on the site. Participants then completed a social network interview with a trained interviewer. During the informed consent process, study interviewers had an in-depth conversation with participants about confidentiality and privacy concerns given the study’s use of Facebook data (see section on ethics in data collection below). The social network interview utilized NameGenWeb, an app that gathered and visualized network data using Facebook’s Application Programming Interface (API; that is, a means of querying a site for data). This approach had a number of advantages over traditional approaches to network data collection. When dealing with self-reported networks, the elicitation of indirect ties (that is, whether people in one’s network are known to each other) is often a time consuming but necessary task, given that indirect ties are often of central interest. Researchers have approached this as a series of person-by-person questions [37] and a task where network members are drawn on paper [38], a whiteboard [39], or rendered by a computer [40]. In contrast to these approaches, a theoretical advantage of collecting data from social media is that indirect ties can be queried in a programmatic way. Thus, instead of asking respondents to report on the potential ties between ten or twenty closest personal network members, Facebook might efficiently return the indirect ties between hundreds or thousands of personal network members. As Facebook ties are drawn almost exclusively from offline friendships [4], the use of Facebook to collect network data enabled the rapid collection of an online network in the size and scope of one's personal network with minimal respondent burden. 

**Figure 1:**
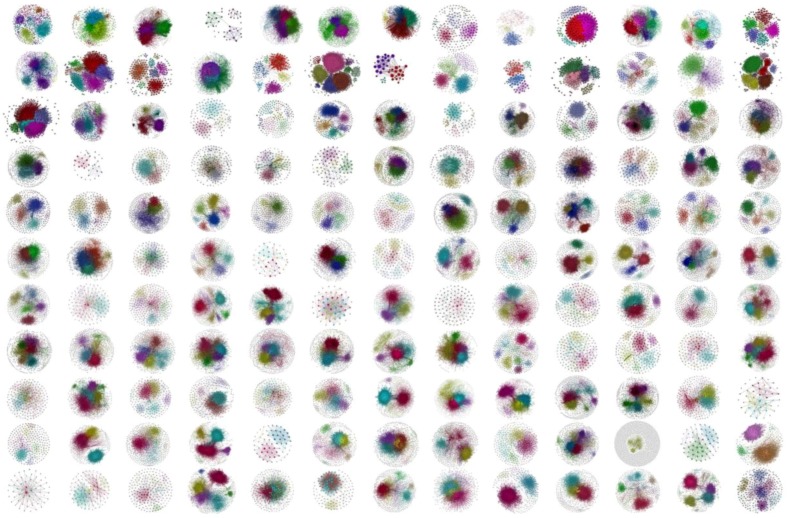
The participants’ individual Facebook networks. *Note:* Each node represents a single Facebook contact, and each edge represents a connection between two Facebook contacts. Network subgroups (as identified by NameGenWeb) are illustrated in different colors.

NameGenWeb generated a visualization of the participant’s network data that utilized a clustering algorithm to identify network subgroups, which were visualized using different colors. Once the visualizer was launched, the interviewer explained the graphic to the participant, indicating that friends were represented in the graphic as dots, that a line between two dots indicated a Facebook friend connection, and that different colors indicated different groups of people based on friendship connections. (For an illustration of these network visualizations, see Figure 1). Each network subgroup was pulled up one at a time on the visualizer, and participants were asked to name each group in a way that described how the members were connected. Participants were also provided with a list of common group categories (family, school, work, church, neighborhood, LGBT community, LGBT family, and other) and asked to select a category for each group named. The interviewer recorded the group name and category for each subgroup identified in the NameGenWeb visualization. 

At the end of the interview, the participant’s Facebook network data was downloaded as a .gexf file, which was subsequently deidentified and stored on a secure server. The NameGenWeb app was uninstalled from the participant’s Facebook account at the end of the interview. Interviews lasted approximately 90 min. Participants were compensated with $50 cash or a $50 gift card mailed to their preferred address.

Five participants were missing data on key variables, resulting in an analytic sample of 199 for the cluster analysis. An additional 75 participants were dropped in the second phase of analysis due to not having an active Facebook account (*n* = 26) or missing Facebook network data files (often due to participants completing interviews remotely; *n* = 49), resulting in an analytic sample of 121 for the network visualization.

### 2.2. Ethics in Data Collection

From 2007 to 2015, Facebook made the ability to query indirect ties available to registered Facebook applications. In 2015, Facebook restricted these data since it was claimed that a small number of companies were abusing this system. In 2018, it was revealed that such abuse had been taking place, with companies such as Cambridge Analytica utilizing data in violation of Facebook’s terms of service. This means that this study, with data collection conducted between 2013 and 2014, represents a rare opportunity to look at the relationships between outness and Facebook network structure for LGBTQ young people. This opportunity also presented a challenge: how can we leverage social network data in a way that is ethically sound rather than assume that because data exists, it exists to be used [41]? Answering this question is not merely philosophical; it helped inform our methodology and limited some of the possible analyses we conducted. 

Given the potential ethical challenges associated with Facebook network data, interviewers had an in-depth conversation about data security and privacy with participants during the informed consent process. Participants were informed that people in their Facebook networks would be considered secondary subjects as information about them would be accessible to study researchers. However, participants were informed that: (1) the study focused on network structure rather than the individuals in their social networks; (2) network data would be deidentified; (3) researchers would only have access to data made available by participants’ and secondary subjects’ privacy settings; (4) the information accessed was similar to information accessed by other third-party applications; and (5) the app would not post to the participant’s Facebook page and would be deleted at the end of the interview. A Frequently Asked Questions document was created based on anticipated questions so study researchers would be able to answer any questions that arose. We discuss our experiences talking with participants about these privacy concerns in greater detail elsewhere [35]. 

The data accessed and visualized by NameGenWeb was shaped by the Facebook API as it existed at the time of data collection [42]. This API enabled an app developer to download friendships among the people the respondent knew, with a few exceptions: if the alter blocked the friend or put the friend on a limited profile, they would not appear in this network. If the alter prevented anyone from seeing the alter’s friend list, this alter would not appear in the network. NameGenWeb, at the time, had a clear privacy policy stating that the app developers themselves did not process any data collected via the app and deleted all data collected via the app. Unlike NameGenWeb (or its contemporary NetVizz [43]), other apps have in fact used this data in questionable ways (such as Cambridge Analytica), captured it in questionable ways (the Harvard Taste, Ties, and Time dataset; [44]) or harvested data to share with other researchers (MyPersonality [45]).

Although researchers have articulated the need for clear guidelines on internet research, these did not exist at the time of the study. The Association of Internet Researchers developed guidelines that suggested discretion without providing strict guidance [46]. The social network community also provided some ethical guidelines on network data collection [47], but the issue of managing indirect consent and secondary subjects remained unresolved [48]. In the absence of clear guidelines, we sought to be as cautious as possible while remaining faithful to the goals of the study.

Our ethics in the process of network data collection and analysis were based on parsimony, data minimization, and confidentiality. *Parsimony* in data collection refers to the fact that the data about friends we collected was the same data that was available in the everyday use of Facebook [49]. Note that collecting data in a manner that conforms to the everyday expectations of research participants is important so as not to upset participants, undermine their trust, or appear invasive. Our use of data about “mutual friends” was consistent with participants’ everyday use of Facebook data. *Data minimization* refers to the collection of data only as was necessary for the study aims. In our case, we were not interested in a respondent’s friends directly. We did not seek other data such as friend’s age, location, and school even if such data was available through the API. Rather, we focused on the friendship connections (that could be seen by the respondent). Even names were only employed as a means to help the respondent establish the role for each of the network subgroups. We did not triangulate data (as was done in the ethically problematic Taste, Ties, and Time data; [44]) or otherwise seek out additional data about alters. Finally, *confidentiality* is an essential part of building trust with the respondent. We used an app that did not keep data and we did not post to a user’s wall nor send messages to alters in the network or distribute the data. Further, we ensured the app was deleted from the participant’s Facebook page prior to the conclusion of the interview and a number of data security procedures were put in place to securely store participants’ deidentified network files.

All participants gave their informed consent for inclusion before they participated in the study. The study was conducted in accordance with the Declaration of Helsinki, and the protocol was approved by the Institutional Review Board at Northwestern University (STU00206043).

### 2.3. Analytic Strategy

To address our first research question, we used cluster analysis to explore patterns of outness to family, classmates, co-workers, and others in general in order to determine if we were able to identify distinct and interpretable cluster types representing a typology of outness for the LGBTQ young people in our sample. We followed a two-step procedure to examine cluster types [50]. First, hierarchical cluster analysis was conducted [51] in SAS 9.4 (Cary, NC, USA). This step allowed us to examine patterns of explained variance in order to determine the cluster solution that best fit our data. Second, we used nonhierarchical k-means clustering with PROC FASTCLUS in SAS 9.4, specifying and evaluating multiple cluster solutions to decide on a final cluster solution. We specified nonhierarchical k-means clustering with two, three, four, and five cluster solutions. We found the four-cluster solution to be the most interpretable and to result in the largest increase in variance explained by cluster type. Results of the hierarchical cluster analysis suggested a four-cluster solution maximizing between-group variability while minimizing within-group variability.

To address our second research question, we aggregated and visualized participants’ Facebook networks by cluster type. We began with the 121 individual networks (see Figure 1) and went through a process of network aggregation and vertex contraction to illustrate patterns in relational subgroups across the four cluster types. First, we took all participants’ ego-centric networks in Cluster 1. Each ego-network represented connections among one participant’s Facebook contacts (alters). Since these alters belonged to certain relational subgroups (for example, family, classmates, and co-workers), the networks also held information on the connectedness of these social groups in each ego’s social network. In order to better understand how social connections among individuals reflect the connectedness of social groups, we contracted each pair of edges (social connection between two alters) in the graph (which represented all ego-centric networks for participants in Cluster 1). As a result, the two nodes (alters) of a contracted edge (connection) were replaced with new nodes in such a way that the new nodes represented the specific attribute of the original nodes along which vertices were contracted. Since our attribute represented network subgroups, all the individual nodes (alters) were replaced by their group membership attribute, and the number of nodes in the new graph equaled the number of relational subgroups. Node size was weighted to show the proportion of the total alters in the aggregated network that belonged to that relational subgroup. Furthermore, individual edges between two social groups were replaced by one weighted edge, where the weight of the inter-group edge equaled the proportion of the total edges in the aggregated network that were found between those two relational subgroups. We weighted the nodes and edges using proportions rather than raw counts in order to account for the different numbers of participants in each outness cluster type. We then repeated the same process for each of the four cluster types. This allowed us to examine and visualize patterns in network composition (the proportion of alters in each relational subgroup) and network connectivity (the proportion of edges between each pair of relational subgroups). 

To address our third research question, we examined the qualitative data from participants’ responses to open-ended questions about how they used Facebook and their positive and negative experiences online. In-depth qualitative data collection was not the primary focus of the study, as participants also completed survey questions and the social network interview. Given the limited number of open-ended questions and the short nature of participants’ responses, we did not have sufficient data for in-depth qualitative analysis. Instead, we identified key quotes related to identity management and outness, which we grouped by cluster type. These key quotes provide some additional context about the nature of participants’ online relational experiences.

## 3. Results

### 3.1. Examining Cluster Types

**Figure 2:**
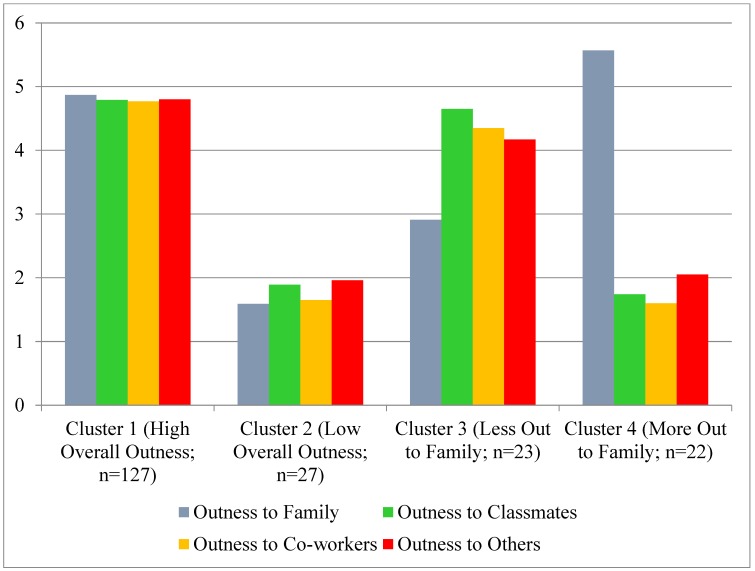
The cluster type means on outness to relational subgroups.

The two-step cluster analysis procedure resulted in a four cluster solution. For a visual depiction of the outness clusters, see Figure 2. 

*Cluster 1—High Overall Outness*. Cluster 1 (*n* = 127; 63.82% of participants) was characterized by high levels of family (*M* = 4.87, *SD* = 0.34), classmate (*M* = 4.79, *SD* = 0.55), co-worker (*M* = 4.77, *SD* = 0.57), and other (*M* = 4.80, *SD* = 0.49) outness. 

*Cluster 2—Low Overall Outness*. Cluster 2 (*n* = 27; 13.57% of participants) was characterized by low levels of family (*M* = 1.59, *SD* = 0.78), classmate (*M* = 1.88, *SD* = 1.18), co-worker (*M* = 1.65, *SD* = 1.06), and other (*M* = 1.96, *SD* = 0.94) outness. 

*Cluster 3—Less Out to Family*. Cluster 3 (*n* = 23; 11.56% of participants) was characterized by lower outness to family (*M* = 2.91, *SD* = 0.67) than to classmates (*M* = 4.65, *SD* = 0.67), co-workers (*M* = 4.35, *SD* = 0.88), and others (*M* = 4.17, *SD* = 0.78). 

*Cluster 4—More Out to Family*. Cluster 4 (*n* = 22; 11.06% of participants) was characterized by higher outness to family (*M* = 4.57, *SD* = 0.60) than to classmates (*M* = 1.74, *SD* = 0.93), co-workers (*M* = 1.60, *SD* = 0.88), and others (*M* = 2.05, *SD* = 1.12). 

### 3.2. Cluster Differences in Facebook Networks

**Figure 3:**
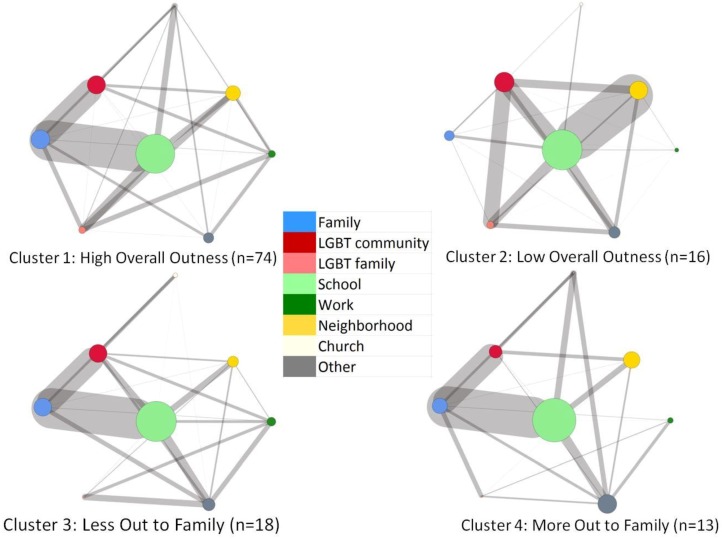
Network composition and connectivity by cluster type. *Note:* Node size is relative to the proportion of alters in each relational subgroup. Edge size is relative to the proportion of edges between each pair of relational subgroups.

We examined cluster differences in Facebook networks with respect to both network composition and network connectivity. The results are reported in Table 1 and Figure 3. Table 1 depicts the proportion of network alters in each relational subgroup across the four cluster types. Figure 3 depicts the proportion of alters in each relational subgroup (illustrated in node size) as well as the proportion of edges between each pair of relational subgroups (illustrated in edge size). With respect to network composition, we found that young people in Clusters 2 (Low Overall Outness) and 3 (Less Out to Family) had the smallest percentage of family members represented in their Facebook networks (7.9% and 10.5%, respectively). Young people in Cluster 1 (High Overall Outness) had the lowest percentage of church alters represented in their Facebook networks (0.7%). In terms of the connection to the LGBT community, young people in Cluster 2 (Low Overall Outness) had the highest percentage of LGBT alters (21.3%), and young people in Cluster 4 (More Out to Family) had the lowest percentage of LGBT alters (11.1%). 

With respect to network connectivity, Cluster 2 (Low Overall Outness) showed the most distinct patterns. While the other cluster types showed high connectivity between school and family and between family and LGBT community, Cluster 2 showed lower connectivity between these network subgroups, but higher connectivity between school and neighborhood. Surprisingly, Cluster 3 showed strong connectivity between LGBT community and family, even though individuals in this cluster type were less out to family than to others in their lives. Finally, Cluster 1 (High Overall Outness) showed the most evenly distributed connections between the network subgroups, as illustrated in its visual appearance as the most complete diamond.

### 3.3. Qualitative Experiences Online

To examine how LGBTQ young people described their experiences on Facebook, we identified key quotes from participants’ qualitative responses to questions about how they used Facebook and their positive and negative experiences online. These quotes are listed separately by cluster type below. 

#### 3.3.1. Cluster 1 (High Overall Outness)

*[I use Facebook] for talking to current friends, maintaining a relationship with old friends, and I will eventually get a separate page for my family to add me on. I just feel bad about having the need to create a second page for simply talking to my family because many of them don’t accept my true well-being, that is, me being happily gay and seeing other people. Embracing and accepting that part of me so openly sometimes upsets them. So this account… is the only place I can truly express myself freely.*

—Black cisgender female, lesbian, age 24

*Some people are anti-gay or anti-feminist and don’t like articles I post, or I’ll argue with them about stuff and then they troll my page. The last time was a White person that didn’t like my anti-racist article and decided to show me “not all White people are bad”, like I don’t already know that.*

—Black cisgender female, lesbian, age 26

*I put up a post with Cinderella and Belle (Disney characters) in an embrace with words on it saying, “My prince charming is a princess”, and several of my “Christian” friends/associates responded saying they would appreciate if I wouldn’t put up pictures depicting Disney characters in such a fashion.*

—Multi-racial cisgender female, questioning/unsure, age 24

*I also use this account to manage multiple “pages”, one of which is for my drag persona. This account has received some negative feedback from more conservative family and friends. They found the overt sexual references and bold personality of a drag show and pictures of it/videos of performances/other marketing materials somewhat offensive. I then added them to the limited visibility privacy group I created to keep people who feel this way from seeing my raunchier posts.*

—White cisgender male, gay, age 26

*[A negative experience was when] I posted a picture of myself and someone posted, “Oh my God, that’s a man”.*

—Black transgender female, gay, age 25

*When I posted a semi-inappropriate pic online recently, my mother wasn’t too fond of it.*

—Black cisgender female, bisexual, age 22

*Ideally, I think it is smart to separate professional colleagues, family, and fellow alumni from my LGBT circle and those I am sexually intimate with.*

—Black cisgender male, gay, age 22

*I have two Facebook pages because I have some younger family members on my main page and I talk about some real nasty freaky things and they don’t need to know about that.*

—Black cisgender female stud, lesbian, age 25

#### 3.3.2. Cluster 2 (Low Overall Outness)

*[I use Facebook] to keep in contact with old friends, or to be nosy because everybody puts their whole life on Facebook… [A negative experience was when] one time I got my nails did and the nails were very pretty but someone commented negatively on the size of my hands.*

—Black transgender female, heterosexual, age 26

*One Facebook account is to keep in touch with all my associates, friends, past coworkers and teachers. One Facebook is to study astrology. One Facebook is to have an alias that I use to ‘like’ my own statuses. One Facebook is one I used to use to document my personal thoughts and feelings, since I couldn’t express them to no one else.*

—Asian/Pacific Islander cisgender male, gay, age 26

*I put a scripture up from the Bible and I got a lot of likes on that post.*

—Multi-racial cisgender male, bisexual, age 24

#### 3.3.3. Cluster 3 (Less Out to Family)

*[I use Facebook for] updates for networking events and volunteer opportunities for things within… the legal community and throughout the law school. And to trick my mom and aunts into thinking they have a peek into my actual life.*

—Black cisgender female, bisexual, age 24

*When I graduated from undergrad, and entered both grad school and the work force, I didn’t want my undergrad raunch to harm my image or my chances of becoming employed. I’m also just uncomfortable with folks in my professional sphere knowing about my personal life and relationships and vacations, etc. So I now have a very limited second personal profile with only a few photos, most of which include myself with professionals in my field or doing volunteer service. No pictures with high/drunk friends, skydiving travel adventures, or food I made. Just the mask I allow employers and my employment network to see... [My other account] is where I will post provocative questions about politics, gender, race, and sexuality… I use it to share interesting things I found online and update about myself.*

—Black cisgender female, bisexual, age 24

*I rarely post about being gay to not discomfort others.*

—Latina cisgender female, lesbian, age 26

*I posted a picture of my partner and I and received a lot of positive comments and likes.*

—White cisgender female, lesbian, age 24

#### 3.3.4. Cluster 4 (More Out to Family)

*[My female partner] and I recently got married, so we’ve been posting some pictures from the day. People are very supportive of our relationship and enjoy looking at our pictures.*

—White transgender female, queer, age 27

*A lot of drama/negative feedback comes from anywhere on Facebook. I currently have females tracking my Facebook page talking down to about me to other friends, family turning on me for something that was out of my hands. My relationship was almost jeopardized.*

—Black cisgender female, bisexual, age 23

As these quotes illustrate, participants reported a range of ways they use Facebook as well as positive and negative experiences online. Some participants used multiple accounts to manage the information that was shared with diverse relational subgroups, particularly information that related to their LGBTQ identities, was sexual in nature, or otherwise reflected behavior they did not want their entire Facebook networks to know about (for example, alcohol or drug use). Work and family were the two contexts participants mentioned most frequently with respect to the need for multiple accounts. Aside from the use of multiple accounts, other participants talked about monitoring or censoring what they post about online out of concern over how others would respond. 

Participants also described a range of experiences with respect to information they shared on Facebook. Some participants received affirming responses related to the LGBTQ-related content they shared, such as “likes” in response to wedding photos. However, participants more commonly identified negative responses, such as transphobic or homophobic comments. These responses illustrate how information shared across diverse relational subgroups can lead to interpersonal conflict and stress for LGBTQ young people. Overall, participants from all cluster types described these identity management strategies and stressors related to context collapse.

## 4. Discussion

Study findings highlight the complex relationships between outness, Facebook network structure, and online experiences for LGBTQ young people. Using cluster analysis, we were able to identify four distinct patterns in how LGBTQ young people manage outness to different relational subgroups. The results showed that most participants reported uniformly high or low outness to all relational contexts, but other participants reported differential levels of outness to family as compared to other people in their lives. Nearly two-thirds (63.8%) of participants fell in the High Overall Outness cluster type, which is heartening given established links between outness and well-being [14] and the likelihood that this pattern reflects LGBTQ young people who feel comfortable disclosing across a range of relational contexts. The high proportion of participants in this cluster type should be understood within the context of the study sample, which was comprised of young adults in an urban setting who had identified as LGBTQ for some time. Given that young people in rural areas experience more negative reactions to coming out [14], the percentage of young people in this cluster type would likely be lower in a rural sample. 

Outness to family emerged as an important variable that distinguished between cluster types, which is consistent with previous research documenting the importance of family as a relational context within which the presence or absence of acceptance and support shapes outness [17,18] and mental health [19,20] for LGBTQ young people. In particular, youth in the Less Out to Family cluster type appear to negotiate outness to relational subgroups in complex ways. These negotiations can be understood as a resilience strategy for minimizing experiences of stigma and discrimination in an effort to preserve psychological well-being [52]. At the same time, if these disclosure negotiations reflect active identity concealment in order to avoid experiences of family conflict or rejection, they are likely to negatively impact psychological well-being [12]. We did not directly examine the level of acceptance and support participants experienced in different relational contexts, and this is an important direction for future research. Additionally, previous research [19] has found that family support increases over time for LGBTQ young people who lack this support early in adolescence. Thus, it is possible that a greater number of participants would have fallen into the Less Out to Family cluster type in a younger sample. 

Interestingly, some young people in the current study reported being more out to family than others in their lives. It is possible that this cluster type reflects individuals who have lower identity salience with respect to their sexual and/or gender minority status and, thus, feel less motivated to share this information across multiple relational contexts. In other words, the young people in this cluster type may view their identities as “not a big deal,” or as personal information that they only share with close network members like family. Participants in this cluster type had the lowest proportion of LGBTQ alters in their networks, which supports this interpretation. This cluster type represents an interesting deviation from the types of negotiations related to outness heretofore examined in research, which typically focus on LGBTQ youth who censor information about their identities from non-affirming families [17,18,26,27].

To our knowledge, this is the first study to examine the Facebook network structure of LGBTQ young people. Our use of NameGenWeb to capture participants’ Facebook networks made this analysis possible and was a unique strength of this study, particularly given the shifting climate around Facebook data sharing and privacy. We have written elsewhere about participants’ experiences of the social network interview [35], where overall participants expressed enjoyment of this portion of the study and identified minimal privacy concerns associated with this approach. The use of NameGenWeb enabled us to capture and analyze Facebook network data in a fraction of the time and with significantly less respondent burden than these processes would have taken using traditional methods. Further, by viewing and discussing the meaning of these networks in interactive interviews with participants, we were able to incorporate participants’ knowledge about these relational contexts into our analysis of network structure. 

With respect to Facebook network composition, school was by far the most popular relational context represented in participants’ networks, accounting for about one-third of the alters across all individual networks. Given that participants were young adults, it is perhaps not surprising that school represented such a large proportion of network alters. LGBT alters (combining both LGBT family and LGBT community) were the next most common, accounting for between 10–20% of network alters, which illustrates the extent to which participants in our study were connected to LGBT relational contexts. Representation of LGBT network alters varied by cluster type: those with uniform levels of high or low outness had the highest proportion, while those who were more out to family had the lowest proportion. This finding belies the stereotype that LGBTQ young people who are closeted are more likely to be isolated from LGBT community and highlights how identity disclosure and relational contexts may be associated in counterintuitive ways [52].

With respect to Facebook network connectivity, visualizations illustrated a number of similarities and differences between cluster types. Consistent with research finding that people with more interconnected relational subgroups tend to feel more connected to their network as a whole [32], we found that LGBTQ young people with uniformly high levels of outness showed the most connectivity between different relational subgroups. Participants in these clusters likely experienced less need to keep their different relational contexts separate, given that their identity disclosure was uniformly high across these different subgroups. By contrast, network connectivity was less evenly distributed between relational subgroups for participants in the other three cluster types, for whom disclosure may be a more complicated process.

For participants in three of the four cluster types (High Overall Outness, Less Out to Family, and More Out to Family), network connectivity was greatest between school and family alters, followed by connectivity between family and LGBT community alters. Although this pattern was consistent with overall higher connectivity for the High Overall Outness cluster type, it was striking and counterintuitive for the Less Out to Family cluster type. Participants in the High Overall Outness and More Out to Family clusters seem to have little reason to keep their LGBT community and family relational contexts separate, as they likely disclose their identities within each of these groups. However, participants in the Less Out to Family cluster type should be motivated to keep their LGBT community and family contexts separate given that these connections increase the risk of identity disclosure to family. Further, given that Facebook connections typically represent offline interactions [4], this finding suggests that these participants are negotiating the overlap of these two relational contexts in their offline lives. This raises a number of questions, such as in what settings this overlap takes place and whether or not these families are aware of the LGBT identities of these network alters (particularly given that these connections likely give them access to the Facebook personae of LGBT alters). Overall, this finding highlights that outness to and contact between relational subgroups may operate in different and counterintuitive ways.

The most visually distinct cluster type was the Low Overall Outness cluster, which showed dramatically lower connectivity between family and other relational contexts as well as dramatically higher connections between school and neighborhood. With respect to family, young people in this cluster type appear to show a more expected relationship between outness and network structure, such that family members are excluded from Facebook networks altogether or are isolated from other relational subgroups when they are included. The connectivity between school and neighborhood may indicate that these young people primarily attended school close to where they lived, such as cases where they attended community college or where the highest level of educational attainment was high school or below. Given research on the negative consequences of outness for sexual minority men of lower socioeconomic status [16], it is possible that participants in this cluster represent more economically marginalized LGBTQ young people relative to the other cluster types. However, the Low Overall Outness cluster also showed the highest connection to LGBT community. In addition to having the highest proportion of LGBT network alters, participants in this cluster type also showed stronger connectivity between the LGBT community and LGBT family and between the LGBT community and neighborhood. These patterns may reflect the importance of LGBT relational contexts for these participants, for whom connection to LGBT family and community may be an important compensatory source of support if their families are not supportive of their identities. 

Although rich in their ability to communicate information about the Facebook relational contexts of LGBTQ young people, these network data do not tell us as much about how participants experience and manage their identities on Facebook. The illustrative quotes identified in the qualitative data provide additional context in this respect. For example, a participant in the High Overall Outness cluster identified that she still felt the need to create a separate Facebook page for family, illustrating how identity management may still be complicated for youth who are highly out but whose relational contexts are not uniformly affirming or accepting. Overall, these identity management strategies support the “lowest common denominator” understanding of how young people choose which information to share with their Facebook networks [4]. Further, young people in this cluster who disclose their identities openly on Facebook may experience negative responses to this disclosure, as in the case of the participant who experienced a negative reaction to her post about a Disney princess. Across all cluster types, participants reported use of identity management strategies (including creating multiple accounts and monitoring their online self-presentation) and identified emotional and relational costs associated with negative responses to online expression of their identities when they did choose to share this information with Facebook network members. These patterns illustrate the complex interpersonal dynamics experienced by LGBTQ young people in social media environments characterized by context collapse.

## 5. Limitations and Future Directions

This study had several important limitations. First, the sample was comprised of young adults who had identified as LGBTQ for at least several years and who lived in a major urban area with multiple opportunities to access LGBTQ resources and community. Study findings would likely be quite different for LGBTQ people of different ages, at different points in the identity development process, and in different regions of the country. Second, given that most of the youth in this study fell in the High Overall Outness cluster type, analysis for the other three cluster types was based on relatively small sample sizes; thus, study findings should be viewed as preliminary and exploratory. Third, our approach to examining the Facebook network structure focused on the cluster level by aggregating individual networks within each cluster type. Given that there was variability in the sizes of the networks in our sample, this means that larger networks were, in a sense, over-represented in the cluster-level patterns. The results may have differed if we had used an individual level approach to comparing network structure. Fourth, Facebook friends accumulate over time, which shapes the opportunity structure for connections to form between diverse relational subgroups; for example, if you move across the country, there is little opportunity for your hometown friends and family to meet your current friends or LGBT community. As other researchers have identified (for example, Reference [32]), this means that some of the assumptions typically made in social network analysis may not hold true for Facebook networks. We did not ask participants in this study about their geographic histories or other factors that could shape this opportunity structure, and this is an important future direction for online social network research. Fifth, our use of Facebook data to examine network structure provides an important complement to largely qualitative research examining LGBTQ young people’s Facebook experiences, but tells us less about the nuances of their Facebook lives, such as their identity management strategies. The key quotes provide some insight into these experiences, but more in-depth examinations of these experiences are warranted. Future research could also explore the potential impacts of network connectivity between diverse relational contexts and alters with conflicting political or religious attitudes, such as whether this connectivity has the potential to lead to attitudinal change or increased polarization. Finally, we were specifically interested in how LGBTQ young people manage their identities on Facebook, but young people are increasingly using a range of other social media platforms, such as Instagram, Snapchat, Grindr, and Tumblr. Given that Facebook uses a “real name” policy with respect to users’ identities, LGBTQ young people likely have very different experiences of their identities and communities on other forms of social media.

## 6. Conclusions

The title of this paper was drawn from one of the illustrative quotes in this study, where a participant said she uses social media to “be nosy because everybody puts their whole life on Facebook”. Ironically, this participant was from the Low Overall Outness cluster type—the type that showed the most distinct patterns with respect to Facebook network composition and connectivity, and for whom the segmentation between family and other relational contexts appears to be the strongest. In fact, it is possible that this participant commented that “everybody puts their whole life on Facebook” precisely because she herself was more careful about what she shares online given potential negative consequences of disclosing her identity, which she went on to describe with respect to transphobic comments about her hands. As this participant’s experience illustrates, study findings show that while some people may indiscriminately share information about themselves on social media, for LGBTQ young people these decisions are infinitely more complicated.

## Figures and Tables

**Table 1:** The Facebook Network Composition by Outness Cluster Type.

Relational Subgroup	Cluster 1 (High Overall Outness; *n* = 74)	Cluster 2 (Low Overall Outness; *n* = 16)	Cluster 3 (Less Out to Family; *n* = 18)	Cluster 4 (More Out to Family; *n* = 13)
Family	14.5%	7.9%	10.5%	11.8%
School	30.0%	31.5%	31.4%	33.5%
Work	5.4%	3.2%	6.8%	4.3%)
Church	0.7%	2.7%	3.7%	0.9%
Neighborhood	11.4%	14.3%	8.5%	12.7%
LGBT Community	14.0%	15.6%	14.0%	9.9%
LGBT Family	5.3%	5.7%	1.5%	1.2%
Total LGBT	19.3%	21.3%	15.6%	11.1%
Other	8.0%	9.0%	9.7%	14.6%
Isolate	10.7%	10.1%	10.5%	11.1%

Note: The percentages reflect the aggregated number of alters in the Facebook networks of participants in that cluster type.
